# The impact of city size on income inclusive growth: A human capital perspective and evidence from China

**DOI:** 10.1371/journal.pone.0288294

**Published:** 2024-02-12

**Authors:** Shao-ling He, Yuan Zhong, Wei-wei He

**Affiliations:** 1 School of Medical Humanities and Management, Hunan University of Medicine, Huaihua, Hunan, China; 2 School of Economics and Trade, Hunan University, Changsha, Hunan, China; 3 School of Economics, Hunan University of Finance and Economics, Changsha, Hunan, China; Qufu Normal University, CHINA

## Abstract

This paper methodically investigates the influence of inclusive income growth on city size, examining it through the dual lenses of "income" and "distribution." The analysis leverages meticulously collected panel data encompassing 276 Chinese cities at the prefecture level and above, spanning the period from 2005 to 2019. Theoretical analysis indicates that the effect of city size expansion on per capita income adheres to a ’U’-shaped trajectory, while its influence on the urban-rural income gap manifests an ’inverted U’ pattern. Moreover, the inclusive income growth stemming from city size demonstrates notable heterogeneity across various geographic locations and city hierarchies. The findings reveal that human capital serves as the primary mechanism through which city size influences inclusive income growth. After decomposing the income inclusiveness index, it becomes evident that the expansion of city size exerts a more potent direct driving effect on the income of urban residents. On the one hand, city size expansion directly increases rural residents’ income levels by improving labor productivity. On the other hand, it facilitates leapfrog income development by inducing the rural labor force to move to cities.

## 1. Introduction

Following the three industrial revolutions, human society has undergone a transformative shift from agricultural to industrial civilization. Within this framework, the swift evolution of industrial structure has propelled the rapid expansion of urban areas, leading to profound alterations in the patterns of residents’ income and welfare. This transformative process is evident in the developmental trajectory of China, a newly industrialized global player. Since the 1980s, China has strategically facilitated the rapid concentration of factors in urban zones through the implementation of the "favoring cities and modern sectors" development strategy. This approach has significantly expedited the industrialization process, propelling the national economy to unprecedented levels of growth and advancement. It is noteworthy that China’s per capita nominal GDP surged from 468 CNY in 1980 to approximately 86,000 CNY in 2022, marking an extraordinary increase of over 182 times. However, amidst this economic ascent, the income ratio between urban and rural residents also rose from 2.30 to 2.45, denoting an increment of around 6.5%. These salient statistics illustrate that China’s economic trajectory has indeed soared within the framework of the "city first and countryside later" development strategy. Simultaneously, the escalating issue of urban-rural income inequality underscores a paradox—while the nation’s economic prosperity is evident in the rapid expansion of the national income scale, the positive impact on optimizing income distribution remains notably limited. This suggests that a development model prioritizing "efficiency" at the expense of "fairness" may face challenges in sustaining itself over the long term. The rationale behind this phenomenon can be elucidated as follows: Drawing upon H. Chanery’s classic dualistic structure theory, during the initial stages of economic development, cities emerge as focal points, attracting a concentration of factors that foster agglomeration effects. This process becomes a pivotal mechanism for regions to drive economic expansion and industrialization. However, as the economy advances beyond a certain threshold, further intensifying the concentration of factors in cities may diminish market allocation efficiency, thereby hindering overall economic growth. In essence, while the unbalanced growth model favoring cities may yield notable short-term results, achieving sustainable growth necessitates breaking away from the dual economic structure and fostering the integration of urban and rural economies. This integration provides a long-term foundation for sustained economic development. In recent years, a discernible and persistent downturn in marginal output within China’s urban sector has become increasingly evident. The conventional development model is poised to encounter heightened impediments as it relies on guiding factors towards urban agglomeration to stimulate growth and enhance the national welfare. Consequently, it is an inexorable imperative for China to urgently delve into an inclusive income growth trajectory that is strategically synchronized between urban and rural areas and mutually reinforcing between industry and agriculture.At the crux of inclusive income growth lies the integral amalgamation of efficiency and equity, as elucidated by [1, 2]. This perspective resonates closely with the tenets of "long-term balanced growth" in traditional dual economic development theory. As China’s economy transitions into a novel developmental paradigm, the emphasis on inclusive income growth that meticulously balances "efficiency" and "fairness" assumes paramount significance. Such a approach holds the key to amplifying domestic demand, propelling high-quality development, and advancing the overarching goal of common prosperity.

In the realm of economics, the dynamics of city size emerge as a focal point, exerting a profound influence on China’s economic metamorphosis. Over the span of 1980 to 2022, the urban demographic share in China burgeoned from 13.9% to 65.2%, with most cities witnessing a rapid demographic ascent. This demographic shift unfolds against the backdrop of seismic changes in the national economy, marking a transformative trajectory in both China’s population distribution and the scale of its urban centers.Extant scholarly investigations posit that while the expansion of city size serves to optimize the efficiency of resource allocation across factors, thereby fostering an upswing in labor income [3], it concurrently exacerbates the urban-rural income disparity [4]. The evolving interplay between China’s urbanization and economic dynamics underscores the intricate challenges and opportunities embedded within the complex tapestry of its economic landscape. By combining pertinent research perspectives with salient empirical data from China’s economic landscape, it becomes evident that the substantial proliferation of city-size expansion initiatives since the advent of economic reforms and opening up has wielded an optimizing impact on the income and welfare of Chinese residents, primarily manifesting in enhanced "efficiency." However, its positive influence on bolstering "fairness" appears relatively circumscribed. In essence, the large-scale expansion of city size in China, propelled by urban-centric industrial policies, tends to prioritize "efficiency" over "fairness." This unbalanced emphasis results in a failure to achieve inclusive income growth, as the overarching strategy lacks a holistic consideration of both efficiency and fairness. This leads us to a pivotal query: Can the trajectory of city size expansion concurrently embody characteristics of being both "efficient" and "fair"? This question underscores the nuanced challenge of reconciling the dual imperatives of efficiency and fairness within the context of China’s urbanization strategy.

In recent years, an expanding body of research has underscored the pivotal role of city size in shaping regional growth [5, 6] and delineating the contours of the urban-rural income gap [7, 8] with marked nonlinear characteristics. While extant studies have yet to offer a comprehensive resolution to the intricate interplay of "efficiency" and "fairness" associated with city size, their consensus on the "nonlinear influence" of city size on both "growth" and "distribution" represents a novel avenue for addressing the challenges posed by "efficiency" and "fairness" within the expanding ambit of city size dynamics. This emerging perspective opens up new avenues for navigating the complexities of efficiency and fairness amid the ongoing expansion of city size. Hence, a thorough and systematic exploration of the question "How to attain inclusive income growth amidst the burgeoning expansion of city size" within this paper not only contributes to the enhancement of pertinent research frameworks but also holds pragmatic significance in refining the governance mechanisms associated with city size and fostering inclusive income growth. In contrast to existing research, the potential marginal contributions of this paper can be delineated as follows:

In the first place, this paper undertakes an analysis based on the dual dimensions of "efficiency" and "equity," probing the tangible influence of city size on income-inclusive growth. Existing studies often present unilateral discussions either on the "efficiency" or "equity" impacts of city size, thus inadequately capturing the comprehensive transformation in residents’ income and welfare amidst the evolving urban landscape. By systematically scrutinizing the inclusive income growth effect of city size through the lenses of both "efficiency" and "equity," this paper not only supplements and enhances the existing research paradigm but also contributes significantly to a deeper comprehension of the nexus between city size dynamics and inclusive income growth.

Secondly, leveraging a nonlinear perspective, this paper extensively delves into the implications of city size on inclusive income growth, employing pertinent data from prefecture-level and higher cities in China. In contrast to prevailing studies focused on the "city size and resident income" discourse primarily at transnational or provincial levels, this paper pioneers the use of meticulously curated balanced panel data spanning 276 cities at the prefecture level and above in China, covering the period from 2005 to 2019. Through this, the research investigates the non-linear impact of city size on inclusive income growth. Such a nuanced approach not only contributes to the augmentation of the current research framework concerning city size and income-inclusive growth but also furnishes empirical substantiation for policymaking in the domain of city size and inclusive income growth governance.

The subsequent sections of this paper are organized as follows: The second section encompasses a comprehensive review of pertinent literature; the third section delineates the configuration of the empirical model and provides a detailed account of the utilized data; the fourth section is dedicated to the empirical testing and subsequent analysis of results; the fifth section involves the examination and analysis of the underlying mechanisms at play; the sixth section delves into the expansive dynamics of city size and inclusive income growth through the lens of index decomposition; finally, the seventh section encapsulates the conclusions drawn from the study and proffers policy recommendations.

## 2. Literature review

### 2.1 City size and inclusive income growth

Current research places relatively less emphasis on the direct impact of inclusive income growth concerning urban scale, instead focusing more on the consequential influence of urban scale on regional development. Within this realm, mainstream investigations underscore that the expansion of urban centers engenders a labor agglomeration effect, optimizing labor allocation, expediting the diffusion of technology, fostering knowledge dissemination, and augmenting the availability of intermediate goods. Consequently, this catalyzes a substantial impetus for regional growth [9–11]. Nevertheless, in recent years, a burgeoning body of research has identified that the unwarranted enlargement of urban centers can give rise to a "crowding effect." This effect manifests in heightened transportation costs, exacerbated environmental pollution, diminished efficacy in the provision of public services, and the erosion of local factor allocation efficiency, ultimately impeding local economic growth [12, 13]. Moreover, additional research indicates that the influence of urban scale on regional growth may exhibit non-linear characteristics. Henderson (2003), and Chen and Zhou (2017) discerned, through empirical studies grounded in international and Chinese practical experiences, that city size exerts a non-linear impact on regional growth [5, 14].

The findings of extant studies regarding the income distribution impact of city size remain contentious. While some research posits that city size exacerbates the urban-rural income gap [4, 15, 16], divergent perspectives exist. This phenomenon arises due to the agglomeration effect engendered by the expansion of city size, wherein the productivity growth in urban areas significantly outpaces that in rural areas. Consequently, the income of urban residents experiences a swifter ascent compared to their rural counterparts, thus contributing to a widening income gap. Nevertheless, certain studies underscore that the enlargement of city size can mitigate the urban-rural income gap [17–19]. The potential rationale behind this phenomenon lies in the influx of surplus rural labor to urban areas, enhancing the marginal productivity of labor through an improved allocation of factors in the agricultural sector. This, in turn, propels the escalation of sectoral wages, directly narrowing the income disparity between urban and rural residents. Furthermore, the migration of surplus rural labor to the urban sector for employment facilitates substantial advancements in their income levels, resulting in a leapfrog growth pattern. Consequently, this dynamic contributes to a noteworthy alteration in the urban and rural income distribution paradigm. Recent research has unveiled that the influence of city size on the income gap might exhibit a non-linear relationship [7, 8, 20, 21]. According to pertinent studies, during the initial phase of city size expansion, the migration of surplus rural labor to urban areas fosters a labor agglomeration effect within urban sectors. This effect stimulates accelerated growth in labor productivity and income levels within urban sectors, leading to an expansion of the urban-rural income gap. As city size expansion progresses, the diminishing impact of the labor agglomeration effect within urban sectors becomes evident. Further enlargement of city size gives rise to a "crowding effect," which in turn diminishes the marginal productivity of the labor force and the income levels within departments. The agricultural sector’s productivity is expected to persistently increase as surplus labor continues to migrate from the sector. This phenomenon leads to a substantial enhancement in the income levels of rural residents, thereby frequently resulting in a narrowing trend in the urban-rural income gap.

In conclusion, existing studies reveal significant debates regarding the growth impact of city size and its influence on urban-rural income distribution. Given that China’s urbanization rate has surpassed 60% since 2019, the conventional approach of prioritizing cities may exhibit constrained real contributions to local output and potentially impede the progression of urban-rural regional integration. Hence, a timely recalibration of industrial development strategies, coupled with the concurrent pursuit of both "efficiency" and "fairness," becomes imperative to foster China’s high-quality development and propel the indispensable goal of achieving common prosperity.

Contemporary research, grounded in a linear perspective, predominantly accentuates the "agglomeration effect" associated with city size expansion. However, it tends to overlook the conceivable "crowding effect," thus inadequately capturing the comprehensive influence of city size on inclusive income growth. Consequently, this paper posits Hypothesis 1: City size exhibits a non-linear impact on inclusive income growth.

### 2.2 City size and human capital

Human capital has played a constructive role in advancing income growth and mitigating income disparities. Its efficacy in promoting inclusive income growth has garnered unanimous acknowledgment in pertinent studies [22, 23]. The influence of city size expansion on human capital is likely to be intricate. Certain studies have indicated that the enlargement of city size exhibits a skill preference [24, 25]. This preference not only facilitates the rapid migration of skilled labor from rural areas to cities but also incentivizes workers to augment their human capital investments [26]. Consequently, this dynamic is anticipated to expedite the accumulation of local human capital. Nevertheless, certain studies underscore that during the initial phases of economic development, the migration of skilled foreign labor into urban areas fosters agglomeration effects. These effects include optimizing labor allocation, facilitating technology spillovers, and fostering knowledge dissemination. Consequently, the expansion of city size propels the accelerated accumulation of human capital within this progression. Nonetheless, as the urban population size continues to expand, the agglomeration effect of labor factors experiences a gradual decline. Simultaneously, the emergence of a "crowding effect" stemming from exacerbated environmental pollution, elevated transportation costs, and diminishing efficiency in public service supply poses constraints on the continued influx of population [27]. This, in turn, has the potential to impede the ongoing accumulation of human capital. Building upon the pivotal role of human capital in fostering inclusive income growth and considering the intricate impact of city size expansion on human capital, this paper advances Hypothesis 2: Human capital serves as the mechanism through which city size influences inclusive income growth.

## 3. Empirical model setting and data description

### 3.1 Model design

In formulating a basic empirical model featuring quadratic terms of explanatory variables, this study seeks to address the shortcomings of prior research, which predominantly fixates on the one-sided attention of the growth and distribution effects associated with city size. Consequently, this paper endeavors to amalgamate growth and distribution considerations within a cohesive framework, aiming to evaluate the influence of city size on inclusive income growth, as follows:

$$income_{it}=\alpha +\beta_{1}size_{it}+\beta_{2}size_{it}^{2}+\lambda D+\mu_{i}+\delta_{t}+\varepsilon_{it}$$ (1)

$$gini_{it}=\alpha +\beta_{1}size_{it}+\beta_{2}size_{it}^{2}+\lambda D+\mu_{i}+\delta_{t}+\varepsilon_{it}$$ (2)

Where *income* is the local income level, *gini* reflects the urban-rural income gap, the *size* represents the size of cities at the prefecture level and above, and D is the set of control variables at the city level. *μ* and δ are respectively the fixed effect of the prefecture-level and above cities in China (hereinafter referred to as the city effect) and the fixed effect of the year, and *ε* is the random disturbance term. Eq (1) endeavors to illustrate the extent to which the enlargement of city size has attained "efficiency" by capturing the genuine contribution of city size to regional growth. Meanwhile, Eq (2) aims to assess the genuine influence of city size on the disparity in income between urban and rural areas. In essence, it seeks to ascertain whether city expansion corresponds to "fairness" in addressing this income gap.

### 3.2 Variable selection and description

#### 3.2.1 Explained variables

Divergent viewpoints exist in current literature regarding the formulation of an assessment framework for inclusive income growth. Building upon the research conducted by Zhang and Wan (2016) [22], this study opts for the natural logarithm of regional per capita disposable income (*income*, unit: 10,000 CNY) and the urban-rural Gini coefficient (*gini*) as the explanatory variables. These variables are employed to scrutinize the ramifications of fiscal decentralization on both "growth" and "distribution" issues. The computation approach for disposable income aligns with the methodology outlined by Ma et al. (2018) [28]. The specific formula is as follows:

$$income=income_{u}•p_{u}+income_{r}•p_{r}$$ (3)

Income refers to the per capita disposable income of urban and rural residents at the prefecture level and above, income_u_ and income_r_ respectively refer to the per capita disposable income of urban residents and the per capita net income of rural residents, respectively. The *P_u_* represents the proportion of urban population in total population, the *P*_*r*_ represents the proportion of rural population in total population, an *P_u_*+*P_r_* = 1. Furthermore, prevailing empirical analyses concerning Chinese cities at the prefecture-level and above predominantly utilize the urban-rural income ratio as a metric to gauge the disparity between urban and rural incomes. However, this metric solely captures the comparative income levels of urban and rural inhabitants, failing to directly convey changes in the relative population proportions of urban and rural residents in China over the past four decades. Consequently, this paper opts to employ the urban-rural Gini coefficient of cities at the prefecture-level and beyond as the benchmark for assessing the urban-rural income gap. This choice aims to offer a more comprehensive portrayal of the urban-rural income gap in China amid the rapid urbanization process. The calculation method of Gini coefficient in urban and rural areas is also referred to Ma et al. (2018) [28]. The specific formula is as follows:

$$gini=1-p_{r}•w_{r}-p_{u}•\left(2-w_{u}\right)$$ (4)

*p_u_* and *p_r_* respectively represent the proportion of urban and rural permanent residents in the total population. *w_u_* and *w_r_* are the proportion of urban disposable income (unit: 10,000 CNY) and rural net income (unit: 10,000 CNY) to regional total income respectively. The gini value ranges from 0 to 1, a smaller Gini coefficient signifies a narrower disparity in income between urban and rural areas.

#### 3.2.2 Explanatory variables

The majority of extant studies gauge a city’s magnitude using metrics such as the proportion of its population at the prefecture level or above, or the municipal district’s area relative to the total area. However, when evaluating actual city size in regions characterized by early industrialization but substantial population outflow, relying solely on the proportion of the municipal district’s total area might introduce distortions. In line with the approach delineated by Liang et al. (2015) [29], this paper adopts the logarithm of the permanent city population in cities at the prefecture level and above (units: 10,000 people) as the benchmark for measuring city size.

#### 3.2.3 Control variables

Industrial structure has a significant impact on both "efficiency" and "fairness" [30], in this paper, industrial structure(*structure*) is delineated by the ratio of tertiary value added to secondary value added. Notably, financial development (*finance*) exerts a substantial influence on inclusive income growth by modulating household income and distribution, primarily through alterations in the accessibility of financial resources [31]. In this paper, the annual per capita loan balance of financial institutions is reflected (unit: 10,000 CNY/person). The supply of public services has a significant impact on inclusive growth [32, 33]. In this paper, transport infrastructure (*road*), public medicine (*medicine*), and internet development (*internet*) are selected to reflect the supply level of local public services. Transportation infrastructure is reflected by the per capita road area (unit: square meters/person), the per capita number of hospital beds for public medical treatment (unit: beds / 10,000 people), and the development of the Internet is reflected by the proportion of Internet users in the total population (unit: households / 10,000 people). In addition, Environmental regulation (*regulation*) has a significant impact on the development of local industries, which not only directly affects local growth [34], but also leads to significant changes in the development of income distribution [35]. Concerning the research of Ye et al. (2018) [36], this paper constructs regional environmental regulation indexes including per capita industrial soot emissions (unit: ton / 10,000 people), per capita industrial wastewater emissions (unit: ton /1 person), and per capita sulfur dioxide emissions (unit: ton / 10,000 people) for the treatment of three types of pollutants, and then takes the corresponding number to reflect the intensity of regional environmental regulations.

### 3.3 Descriptive statistics of data sources and variables

The foundational data concerning inclusive income growth in this research are extracted from meticulously collected and compiled annual statistical communique and government work reports of Chinese cities at the prefecture level and above spanning the period from 2005 to 2019. Additionally, information regarding city size and control variables is sourced from the China City Statistical Yearbook. Configured for a balanced panel data approach, this study systematically excluded data points with missing or incomplete information concerning both the above-ground level and city. The final dataset comprises comprehensive information from over 276 localities, encompassing various Chinese cities. Moreover, given that comprehensive and systematic statistics on inclusive income growth are available only from 2005 to 2019, the empirical research period for this paper spans from 2005 to 2019. Descriptive statistics of the data are presented in Table 1.

**Table 1:** Descriptive statistics of variable data.

VARIABLES	Ob	Mean	Std.	Max	Min
income	4140	9.90	1.62	69.44	16.67
gini	4140	0.07	0.00	0.61	0.20
size	4140	0.67	2.91	8.05	5.88
structure	4140	0.50	0.13	9.48	0.91
finance	4140	10.06	0.16	162.76	6.12
road	4140	5.65	0.00	73.04	4.32
medicine	4140	17.74	8.14	141.00	39.53
internet	4140	0.18	0.00	3.66	0.17
regulation	4140	1.01	0.77	9.76	3.67

Calculated and sorted by the author.

## 4. Empirical test and result analysis

### 4.1 The basic model test results of city size influencing inclusive income growth

This paper seeks to investigate the correlation between city size and local growth as well as the urban-rural income gap across 276 Chinese cities at or above the prefecture level from 2005 to 2019. In essence, it explores whether the expansion of cities achieves a harmonious equilibrium between "efficiency" and "equity" in economic growth. Employing both linear and nonlinear analytical frameworks, this study examines the impact of inclusive income growth on city size, with the corresponding test results detailed in Table 2.

**Table 2:** Basic model test results.

VARIABLES	income	gini	income	gini	income	Gini
	(1)	(2)	(3)	(4)	(5)	(6)
size	15.3162***	-0.0009	-46.9768***	0.1835***	-14.8504***	0.2506***
	(0.5533)	(0.0064)	(3.6055)	(0.0434)	(3.3486)	(0.0465)
size^2^			5.4216***	-0.0160***	1.9247***	-0.0228***
			(0.3104)	(0.0037)	(0.2950)	(0.0041)
structure					-0.1053	0.0075***
					(0.1316)	(0.0018)
finance					0.0000***	0.0000***
					(0.0000)	(0.0000)
road					0.2214***	-0.0003
					(0.0201)	(0.0003)
medicine					0.0502***	-0.0001
					(0.0063)	(0.0001)
internet					1.3964***	-0.0137***
					(0.3320)	(0.0046)
regulation					0.1140***	-0.0007
					(0.0337)	(0.0005)
Constant	-73.4565***	0.2036***	102.9183***	-0.3185**	31.5834***	-0.4774***
	(3.2560)	(0.0378)	(10.5719)	(0.1272)	(9.6429)	(0.1340)
Year Effect	YES	YES	YES	YES	YES	YES
City Effect	YES	YES	YES	YES	YES	YES
Ob	4,140	4,140	4,140	4,140	4,140	4,140
R-squared	0.9494	0.8610	0.9531	0.8616	0.9653	0.8633

Standard errors are in parentheses.

*** p<0.01

** p<0.05

* p<0.1.

Columns (1)–(2) depict the outcomes of the linear correlation tests between city size and inclusive income growth. In Column (1), the observed effect of city size on per capita income is notably positive and statistically significant, aligning with the research findings of Bradshaw and Fraser (1989), Combes et al. (2012), Li and Cheng (2022) [9–11]. The findings in Column (2) reveal that the influence of city size on the urban-rural income gap lacks statistical significance, diverging from the research conclusions drawn by Sulemana et al. (2018), Yuan et al. (2020), Yao and Jiang (2021) [4, 15, 16]. This implies that, grounded in the linear perspective and accounting for both year and city effects, the enlargement of city size can indeed lead to a substantial enhancement in income levels. However, its impact on mitigating the urban-rural income gap appears to be constrained.

Columns (3)–(6) exhibit the nonlinear test outcomes concerning city size and inclusive growth. In columns (3)–(4), the principal coefficient associated with city size for per capita income is notably negative, whereas the secondary coefficient is significantly positive. This suggests a "first negative and then positive" trend in the influence of city size on regional income levels, aligning with the nonlinear perspective advocated by Henderson (2003), and Chen and Zhou (2017) [5, 14]. The rationale is as follows: during the initial phase of urban expansion, owing to the relatively low industrialization of the urban sector, a substantial influx of unskilled labor occurred, consequently diminishing the productivity of the urban sector and causing a decline in per capita income. As city size continued to grow and urban sectors and industries underwent transformation and advancement, per capita income gradually began an upward trajectory. Concerning the urban-rural Gini coefficient, the primary coefficient associated with city size demonstrates a significant positive correlation, while the secondary coefficient exhibits a significant negative correlation. This pattern suggests a progression followed by a limitation in the impact of city size expansion on the urban-rural income gap, in line with the research findings of Wu and Rao (2017), Castells‐Quintana (2018), He and Zhang (2022), and Cheng et al., (2023) [7, 8, 20, 21]. The control variables are subsequently incorporated into columns (5)–(6), revealing that the signs and statistical significance of the initial and secondary coefficients related to city size remain consistent with those observed in columns (3)–(4). Synthesizing these findings with the earlier conclusions, it can be deduced that the influence of city size on residents’ income follows a "first negative and then positive" trajectory, whereas its effect on the income gap demonstrates a "first positive and then negative" pattern.

Regarding control variables, the industrial structure appears to be less conducive to enhancing residents’ income and, in fact, contributes to widening the urban-rural income gap. This outcome may be attributed to the current limited contribution of China’s tertiary industry to the overall economy. While financial development elevates residents’ income levels, it concurrently exacerbates the income disparity between urban and rural areas. This stems from the fact that despite enhancing the overall income of residents by augmenting their financial accessibility, the financial sector predominantly operates within urban domains at this juncture. Consequently, financial development predominantly bolsters the financial accessibility of urban residents, thereby contributing to continual income escalation in both urban and rural settings. Infrastructure, public healthcare, and environmental regulations play a substantial role in fostering income growth, yet their impact on the urban-rural income gap is not statistically significant. This can be attributed primarily to the disparate allocation of infrastructure, public healthcare, and other resources between urban and rural areas, impeding their efficacy in regulating the urban-rural income gap. Furthermore, environmental regulations predominantly focus on urban sectors, yielding "positive benefits" by promoting industrial upgrading while also generating "negative benefits" through constraints and elimination of polluting industries. Given China’s ongoing economic transition, the "positive effect" of these regulations is effectively counterbalanced by their "negative effect." Consequently, this equilibrium potentially leads to restrained alterations in urban residents’ income, thus exerting minimal impact on the urban-rural income gap. The evolution of the Internet not only positively contributes to narrowing the urban-rural income gap but also directly stimulates per capita income growth. This is attributable to the comprehensive enhancement of residents’ information access facilitated by Internet development, particularly benefitting rural residents. The Internet’s robust influence effectively diminishes the "digital divide" between urban and rural areas, exerting a substantial role in fostering economic equality.

**Fig 1:**
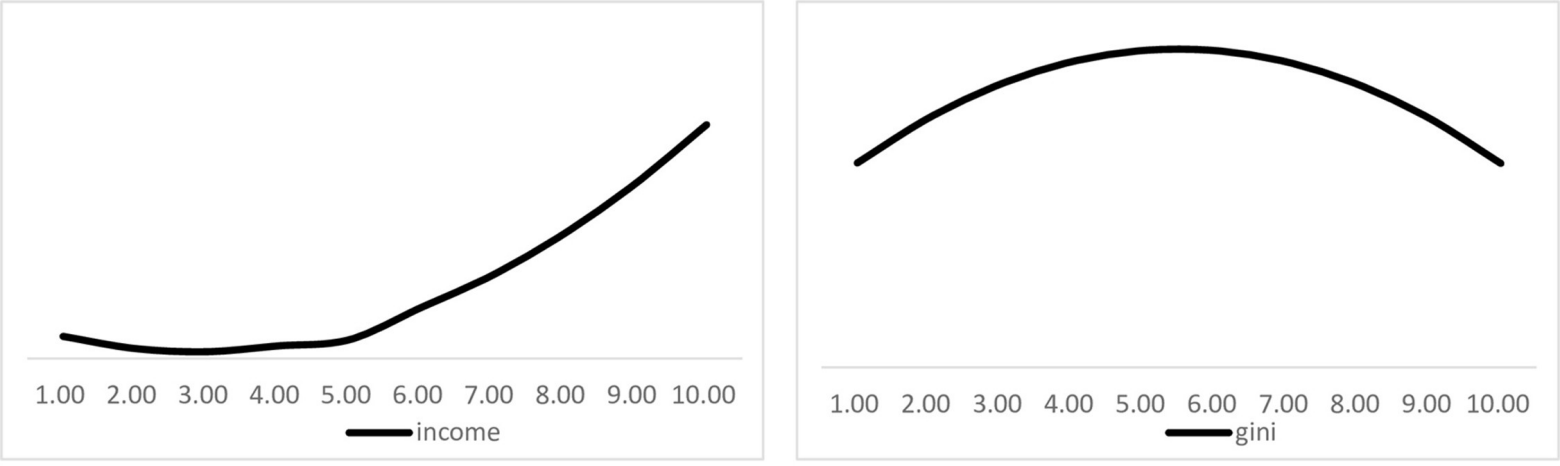
The effect trend of city size on per capita income and urban-rural income gap. Note: In Fig 1, the horizontal coordinate is the logarithm of the city population.

Furthermore, the first-order partial derivative of column (5) about city size can be obtained: ∂income∂scale=3.85scale−14.85. If we set it to 0, we have: *scale* = 3.86, that is, when the city population is less than 473,600, city expansion is not conducive to increasing residents’ income, otherwise it is conducive to the increase of residents’ income. The first-order partial derivative of the city size can be obtained from Eq (6) as follows: ∂income∂scale=−0.05scale+0.25. If we set it to 0, we have: *scale* = 5.50. That is, when the city population is less than 2.4362 million, the city expansion will increase the urban-rural income gap, otherwise it will decrease the urban-rural income gap. The effect of city size on inclusive income growth is roughly shown in Fig 1. It can be found that the impact of urban size expansion on per capita income is "U-shaped", while the impact on urban-rural Gini coefficient is "inverted U-shaped".

The evolution trend of the inclusive income growth effect of city size is shown in Table 3. Drawing from the aforementioned findings, we systematically delineate the evolutionary pattern of inclusive income growth amid the backdrop of urban size expansion, as presented in Table 3. Analysis of the results in Table 3 reveals that when the city population surpasses the threshold of 2,436,200, city expansion can effectively address the dual objectives of augmenting residents’ income and diminishing the urban-rural income gap. Notably, as of 2019, the majority of China’s prefecture-level and higher-tier cities boast populations exceeding this critical threshold. Consequently, ongoing urban expansion initiatives have substantively contributed to fostering inclusive income growth across China.

**Table 3:** Evolution trend of inclusive income growth effect of city size.

The city population	(0,47.36)	(47.36,243.62)	(243.62,+∞)
Income of resident	decrease	increase	increase
Urban-rural income gap	increase	increase	decrease

### 4.2 Robustness test

The outcomes of the robustness test are presented in Table 4. Columns (1) to (2) depict the test results subsequent to substituting the dependent variables. In column (1), where the logarithm of GDP per capita is chosen as a replacement for disposable income per capita, the initial coefficient for city size exhibits a significant negative association, while the second coefficient demonstrates a significant positive relationship. This suggests that the influence of city size on the logarithm of per capita output follows a U-shaped pattern, aligning with the findings of the baseline model. In column (2), the logarithm of the absolute difference between urban residents’ income and rural residents’ income replaces the urban-rural Gini coefficient. The outcomes reveal a notably positive significance for the main coefficient of city size, accompanied by a significant negative value for the secondary coefficient. In essence, the impact of city size on the logarithm of the income disparity between urban and rural residents exhibits an "inverted U" shape, mirroring the outcomes of the foundational model. Columns (3) to (4) present the findings following the substitution of explanatory variables. In this study, the lag period of city size replaces the original explanatory variables, revealing that the initial and secondary coefficients of city size concerning per capita income and the urban-rural Gini coefficient align with those of the foundational model.Columns (5) to (6) unveil the test outcomes subsequent to adjusting for control variables. Through the examination of results after lagging each control variable by one period, it becomes evident that the impact of city size on per capita income and the urban-rural income gap is significantly characterized by a U-shaped and inverted U-shaped pattern, respectively, corroborating the findings of the fundamental model. Columns (7) to (8) present the test outcomes subsequent to the exclusion of specific samples. In contrast to other municipalities, cities directly under the central government exhibit more pronounced advantages in terms of economic size, resource endowment, and policy preference. Consequently, to mitigate estimation bias arising from samples of municipalities directly under the Central Government, we omitted the data from Beijing, Tianjin, Shanghai, and Chongqing and re-performed robustness tests. The results indicate that the parameter signs and significance of the pertinent explanatory variables remain consistent with those observed in the fundamental model.

**Table 4:** Robustness test results.

VARIABLES	income	Gini	income	gini	income
	(1)	(2)	(3)	(4)	(5)
size	-7.3806***	1.3728***	-17.1791***	0.2420***	-12.7601***
	(1.7767)	(0.1895)	(3.5091)	(0.0481)	(3.5730)
size^2^	1.0555***	-0.1248***	2.1883***	-0.0207***	1.6625***
	(0.1565)	(0.0167)	(0.3081)	(0.0042)	(0.3160)
Control Variables	YES	YES	YES	YES	YES
Year Effect	YES	YES	YES	YES	YES
City Effect	YES	YES	YES	YES	YES
Ob	4,140	4,140	3,864	3,864	3,864
R-squared	0.9581	0.9789	0.9658	0.8715	0.9669
VARIABLES	gini	income	gini	income	gini
	(6)	(7)	(8)	(9)	(10)
size	0.2712***	-13.3269***	0.2731***	-78.1206**	2.0098***
	(0.0499)	(3.3541)	(0.0490)	(35.6371)	(0.7763)
size^2^	-0.0239***	1.7713***	-0.0249***	7.4131**	-0.1627**
	(0.0044)	(0.2969)	(0.0043)	(3.0873)	(0.0653)
Control Variables	YES	YES	YES	YES	YES
Year Effect	YES	YES	YES	YES	YES
City Effect	YES	YES	YES	YES	YES
Sargan test				0.421	
Hansen test					0.269
Ob	3,864	4,080	4,080	3,864	4,140
R-squared	0.8710	0.9667	0.8591	-	-

To circumvent potential endogeneity issues in the fundamental model arising from interactions among explanatory and explained variables, potential omitted variables, measurement errors, and other factors, we employed the Systematic Generalized Method of Moments (GMM) to address endogeneity concerns in the baseline model. The test results are illustrated in Columns (9) to (10). In the "efficiency" model of city size, the Sargan statistic of 0.421 signifies the effectiveness of the chosen instrumental variables. After processing the instrumental variables, the impact of city size on per capita income is still "first negative than positive". In the "fair" model of city size, the Hansen value is 0.269, indicating that the selection of instrumental variables is also effective. The impact of city size on the urban-rural income gap is still "first positive and then negative" in the test results after the addition of instrumental variables.

In summary, subsequent to substituting the explained, explanatory, and control variables, along with excluding municipalities directly under central government jurisdiction from the basic model and employing the system GMM method for additional analysis, the parameters associated with the quadratic term for urban per capita income and the urban-rural income gap persist in retaining significance and consistency akin to those in the foundational model. Consequently, the empirical findings indicate stability in the test outcomes of the basic model. Hence, Hypothesis 1 stands confirmed.

### 4.3 Heterogeneity test

The heterogeneity test outcomes are delineated in Table 5. Given China’s extensive geographical expanse, substantial disparities exist in economic development across regions owing to factors such as resource endowments, transportation infrastructure, policy backing, historical antecedents, cultural variations, among others. Hence, the discourse on the heterogeneity concerning the impact of city size on inclusive income growth assumes particular significance. This study endeavors to initially scrutinize inclusive income growth concerning city size through the lens of geographic disparities, with corresponding findings detailed in columns (1) to (8) of Table 5.

**Table 5:** Heterogeneity test results.

VARIABLES	income	gini	income	gini	income	gini	income	gini
	(1)	(2)	(3)	(4)	(5)	(6)	(7)	(8)
size	10.4607***	-29.8691***	-0.0068	-0.2655***	4.3117***	2.3330	-0.0029	0.4906***
	(1.6420)	(8.6807)	(0.0174)	(0.0927)	(0.3929)	(2.7970)	(0.0081)	(0.0566)
size^2^		3.3584***		0.0215***		0.1796		-0.0448***
		(0.7100)		(0.0076)		(0.2514)		(0.0051)
Control Variables	YES	YES	YES	YES	YES	YES	YES	YES
Year Effect	YES	YES	YES	YES	YES	YES	YES	YES
City Effect	YES	YES	YES	YES	YES	YES	YES	YES
Ob	1,500	1,500	1,500	1,500	2,640	2,640	2,640	2,640
R-squared	0.9572	0.9579	0.8297	0.8307	0.9757	0.9757	0.8518	0.8563
VARIABLES	income	gini	income	gini	income	gini	income	gini
	(9)	(10)	(11)	(12)	(13)	(14)	(15)	(16)
size	3.5374*	-60.9510***	0.0203	0.0885	4.2545***	-2.0627	0.0044	0.3361***
	(2.0398)	(10.3351)	(0.0161)	(0.0852)	(0.5017)	(3.6455)	(0.0083)	(0.0598)
size^2^		5.1830***		-0.0055		0.5808*		-0.0305***
		(0.8156)		(0.0067)		(0.3320)		(0.0054)
Control Variables	YES	YES	YES	YES	YES	YES	YES	YES
Year Effect	YES	YES	YES	YES	YES	YES	YES	YES
City Effect	YES	YES	YES	YES	YES	YES	YES	YES
Ob	525	525	525	525	3,615	3,615	3,615	3,615
R-squared	0.9639	0.9668	0.9088	0.9089	0.9675	0.9675	0.8390	0.8405

The impact of city size on inclusive income growth in eastern China is shown in columns (1)–(4). For the eastern region, from a linear perspective, city size increases residents’ income but has no obvious effect on the urban-rural Gini coefficient. However, from the nonlinear perspective, city size has a U-shaped influence on per capita income and urban-rural Gini coefficient. A possible reason is that the eastern region has a better economic foundation, a larger population, and a relatively high per capita income. The expansion of city size not only directly stimulates the rise in residents’ income through the agglomeration effect but also corresponds to a heightened level of urbanization in select cities, resulting in a reduced surplus of rural labor force. The agglomeration effect stemming from city size expansion primarily hinges on the influx of migrant workers into urban areas. In locales where the absorption capacity of the indigenous rural population is insufficient, this dynamic contributes to a widening urban-rural income gap. The analysis of the impact of city size on inclusive income growth in central and western China is delineated in columns (5) to (8). For the central and western regions, the impact of city expansion on per capita income mainly presents a positive linear pattern, while the impact on the income gap shows a significant inverted U-shape. The possible reason is that city expansion not only directly increases residents’ income by optimizing the allocation of labor between urban and rural areas, but also rapidly narrows the urban-rural income gap.

In recent years, China has experienced an acceleration in the trend of unbalanced growth, resulting in a discernible divergence in regional economic prowess. Concurrently, cities are transitioning from a paradigm of regional competition to one of regional cooperation. The propagation of industrial division of labor across the nation, with central cities serving as focal points, has increasingly underscored the trend where other cities engage in industrial division of labor in collaboration with the central city. In contrast to non-central cities, central cities typically wield greater economic influence, exerting a substantial impact on the local economy. Consequently, this study categorizes Chinese cities at the prefecture level and above, designating "municipalities directly under the Central Government and sub-provincial cities" as central cities and categorizing the remaining cities as non-central cities. The analysis subsequently delves into the city-level heterogeneity concerning the influence of city size on inclusive income growth.

The analysis of the influence of city size on the inclusive income growth of central cities is documented in columns (9) to (12). In the context of central cities, the size of the city exhibits a substantial impact on both urban-rural Gini coefficients, with its effect on per capita income demonstrating pronounced U-shaped nonlinear characteristics. Notably, as the permanent population of central cities surpasses the threshold of 3.58 million, the enlargement of city size contributes positively to the income levels of residents in central cities. The underlying rationale for this outcome lies in the fact that, although the size of central cities enhances residents’ income, its role in mitigating urban-rural inequality is constrained by the high degree of urban-rural integration within central cities. The repercussions of city size on the inclusive income growth of non-central cities are elucidated in columns (13) to (16). In the case of non-central cities, city size serves as a direct driver for the augmentation of per capita income, while its impact on urban-rural Gini coefficients exhibits a pronounced inverted U-shape. This phenomenon can be attributed to the heightened urban-rural duality in non-central cities, where the influence of labor allocation driven by city expansion not only contributes to the escalation of residents’ income but also facilitates adjustments to urban-rural income disparities.

## 5. Test and analysis of the mechanism of action

As evidenced above, the influence of city size on per capita income exhibits a noteworthy "U-shaped" pattern, while its impact on the urban-rural income gap demonstrates a distinctive "inverted U-shaped" trend. Given China’s rapid urban expansion over the past four decades, what precisely constitutes the operative mechanism underlying the relationship between city size, per capita income, and the urban-rural income gap? Examining the authentic influence of city size on both per capita income and the urban-rural income gap through the prism of human capital can offer a more nuanced comprehension of the actual mechanisms governing inclusive income growth. Extant research indicates that city size fosters the accumulation of local human capital [37]. Furthermore, human capital exerts a substantive impact on regional economic growth [38] as well as the disparities in income between urban and rural areas [39]. Consequently, this paper endeavors to reevaluate the impact on inclusive income growth in cities at the prefecture level and above in China, taking into account the perspective of human capital.

Contemporary research predominantly utilizes various methodologies to gauge the magnitude of local human capital. These approaches often involve metrics like the proportion of highly educated individuals within the local population, the ratio of college students to the total local population, the relative wage scale, and the formulation of a comprehensive human capital index. In line with the comprehensive dataset encompassing cities at the prefecture level and beyond in China spanning from 2005 to 2015, this paper opts to craft a human capital index to assess regional human capital levels. The specific methodologies adopted for this construction are elucidated below:

$$\text{hc}=\frac{\text{primary}\times \text{6}+\text{secondary}\times \text{12}+\text{university}\times \text{16}}{\text{pop}}$$ (5)

"Primary," "secondary," and "university" denote the respective counts of primary, secondary, and university students, while "POP" represents the total population of the region. Building upon the derived calculations, this study subsequently conducts an in-depth examination of the mechanistic pathways influencing inclusive income growth concerning city size. The outcomes of this analysis are presented in Table 6:

**Table 6:** Test results of the mechanism of action.

	(1)	(2)	(3)	(4)	(5)
VARIABLES	hc	income	gini	income	gini
	(1)	(2)	(3)	(4)	(5)
size	0.3243***			-15.4649***	0.2590***
	(0.0929)			(3.3533)	(0.0466)
size^2^				1.9724***	-0.0235***
				(0.2953)	(0.0041)
hc		0.2729***	-0.0030**	0.2428***	-0.0033***
		(0.0907)	(0.0012)	(0.0886)	(0.0012)
Control Variables	YES	YES	YES	YES	YES
Year Effect	YES	YES	YES	YES	YES
City Effect	YES	YES	YES	YES	YES
Ob	4,140	4,140	4,140	4,140	4,140
R-squared	0.6804	0.9634	0.8624	0.9653	0.8636

The findings presented in column (1) reveal a significantly positive parameter for city size. This implies that the impact of city size on human capital is primarily characterized by an "agglomeration effect" rather than a "crowding effect." This observation aligns with the research conclusions of Elvery (2010), Ye et al. (2016), and Zhou et al. (2013) [24–26]. Building upon the outcomes in columns (2) to (3), it becomes evident that human capital has a direct and positive effect on per capita income, concurrently contributing to the reduction of the urban-rural income gap. This result resonates with the research findings of Zhang and Wan (2016) and Wan et al. (2022) [22, 23]. In columns (4) to (5), the base model is augmented by incorporating the human capital variable. This addition further refines the analytical framework. The results reveal that the influence of city size on per capita income follows a pattern of "first negative and then positive." Similarly, the impact on the urban-rural Gini coefficient exhibits a trend of "first positive and then negative." Importantly, the direction and significance of the associated parameters remain in accordance with those observed in the baseline model. Furthermore, the positive and negative orientation, as well as the significance, of the human capital parameters align with the findings in columns (2) to (3), albeit with slightly diminished parameter values. Consequently, drawing upon the outcomes in Column (1), it can be deduced that human capital serves as an intermediary factor in the expansive process of city size, influencing inclusive income growth.

In light of the aforementioned findings, it is evident that human capital significantly contributes to the dynamics of city size impacting both per capita income and the urban-rural income gap. This affirmation provides empirical support for Hypothesis 2.

## 6. City size and the further expansion of inclusive income growth: From the perspective of index decomposition

The preceding study extensively examined the impact of city size on inclusive income growth and its underlying mechanisms. However, the nuanced effects of city size expansion on the income distribution among urban and rural residents remain incompletely elucidated. To comprehensively delineate the internal dynamics of income variation amid urban and rural populations in the context of city expansion, this study further dissects the "efficiency" and "equity" dimensions of income into four specific indicators: per capita disposable income of urban residents, per capita net income of rural residents, the urban-rural income ratio, and the urbanization rate. The detailed results are presented in Table 7.

**Table 7:** Test results based on index decomposition.

VARIABLES	income_u_	income_u_	income_r_	income_r_	income_ratio_	income_ratio_	urban_ratio_	urban_ratio_
	(1)	(2)	(3)	(4)	(5)	(6)	(7)	(8)
size	0.6372***	-0.3311	0.3110***	-0.8767***	0.0121	0.6788	-0.1083***	-0.5540***
	(0.0527)	(0.3462)	(0.0362)	(0.2368)	(0.1271)	(0.8354)	(0.0088)	(0.0572)
size^2^		0.0863***		0.1059***		-0.0594		0.0397***
		(0.0305)		(0.0209)		(0.0736)		(0.0050)
Control Variables	YES	YES	YES	YES	YES	YES	YES	YES
Year Effect	YES	YES	YES	YES	YES	YES	YES	YES
City Effect	YES	YES	YES	YES	YES	YES	YES	YES
Ob	4,140	4,140	4,140	4,140	4,140	4,140	4,140	4,140
R-squared	0.9675	0.9676	0.9518	0.9521	0.6118	0.6118	0.9605	0.9611

Regarding efficiency metrics, the scale of a city exerts a notably positive influence on the disposable income of urban residents. While the relationship between city size and per capita net income among rural residents is theoretically non-linear, the expansion of urban areas also enhances the income of rural inhabitants, attributable to its lower entry barrier or threshold effect.

Nevertheless, from an equity perspective, the tangible effect of city size on the income disparity between urban and rural residents is constrained. In essence, the expansion of cities does not directly mitigate the absolute income gap between individual urban and rural inhabitants. Furthermore, the impact of city size on the urbanization rate exhibits a pronounced U-shaped pattern, signifying a significant influence. Additionally, owing to its lower threshold effect, the expansion of cities fosters an advantageous environment for enhancing the overall level of population urbanization.

Drawing upon the aforementioned conclusions, it can be deduced that, in terms of efficiency, city expansion exerts a more pronounced influence on augmenting the income of urban residents compared to its impact on rural residents. However, in the realm of equity, the direct reduction of the absolute income disparity between urban and rural residents remains a challenge through city expansion alone. One viable solution involves optimizing the allocation of dual labor, enhancing rural labor productivity, and bolstering the income of rural residents. Additionally, strategically guiding the surplus rural labor force toward urban centers can facilitate a substantial leap in income, contributing to a more equitable income distribution.

## 7. Conclusions and policy recommendations

This paper surpasses the constraints inherent in the study of the "income effect of city size" by integrating the dual dimensions of income growth and distribution. It delves into the ramifications of city size expansion on inclusive income growth and its underlying mechanisms, employing both theoretical frameworks and empirical analyses. Set against the backdrop of China’s accelerating urbanization and the ongoing global economic fluctuations, this paper aims to offer fresh perspectives to facilitate China’s pursuit of high-quality development and foster shared prosperity between urban and rural areas. Leveraging panel data from prefecture-level cities and above in China spanning from 2005 to 2015, this study empirically examines the impact of inclusive income growth on city size. The findings reveal a "U" shaped relationship between city size and per capita income in China, while the influence on the urban-rural income gap follows an "inverted U" pattern, demonstrating robustness. Mechanism test results underscore the pivotal role of human capital in mediating the process through which city size influences inclusive income growth.

To expedite the attainment of high-quality development and foster shared prosperity between urban and rural areas, this paper proffers the following policy implications for the governance of city size and inclusive income growth in China:

Firstly, it is imperative to steadfastly embrace urbanization development and stimulate the expeditious growth of the local economy and personal income. The sustained enlargement of population size within confined spaces exerts a significant impact on economic growth and residents’ income, attributable to both the "agglomeration effect" and "crowding effect" [6, 13]. Conclusive findings substantiate that the expansion of city size manifests a conspicuous "U-shaped" influence on growth. Nevertheless, considering that the size of China’s cities has already surpassed the inflection point at the current stage, the expansion activities of various cities play a conducive role in the rapid augmentation of residents’ income. Therefore, the foremost strategy involves dismantling the constraints imposed by urban and rural household registration, fostering the integration of the labor market, directing surplus rural labor force to urban centers, thereby unleashing the full potential of the "agglomeration effect" and propelling substantial growth in residents’ income. Additionally, there is a need to augment the provision of infrastructure and public services, earnestly striving to enhance the "agglomeration effect" and mitigate the "congestion effect" within the framework of city expansion.

Secondly, enhance the income distribution system and mechanisms, actively harnessing the regulatory capacity of city size expansion to narrow the urban-rural income gap. Despite the "inverted U" shaped relationship between city size and the urban-rural income gap, the actual city sizes in most Chinese regions have already exceeded the turning point. Hence, further promoting city size expansion can effectively contribute to diminishing the urban-rural income gap. To accomplish this, firstly, optimizing the efficiency of allocating agricultural resources is essential, accelerating the management of surplus rural labor outflow to bolster rural residents’ incomes. Secondly, ramping up support for rural infrastructure and public service construction is imperative to foster the unified advancement of urban and rural public services. Thirdly, enhancing the income distribution system and actively advocating for "equal pay for equal work" across urban and rural domains is pivotal.

Thirdly, expedite the accumulation of human capital and institute a comprehensive governance framework for managing city size and fostering inclusive income growth. Owing to substantial disparities in economic development, factor endowments, natural conditions, and various dimensions across different regions in China, the correlation between city size and inclusive income growth is intricate. Hence, expediting human capital accumulation and establishing a unified, resilient governance system holds particular significance. To realize this objective, firstly, there is a need to augment investments in public education, actively draw in high-end technical talents, and expedite the establishment of a high-level local human resources system. Secondly, local governments should implement judicious regulation and control of city size, residents’ income, and income distribution tailored to their specific developmental context. Thirdly, it is essential to seamlessly integrate with the local industrial structure, financial development, environmental protection, and other pertinent domains, facilitating multi-dimensional collaborative governance of city size and inclusive income growth.
